# Spotted Fever Group Rickettsioses in Israel, 2010–2019

**DOI:** 10.3201/eid2708.203661

**Published:** 2021-08

**Authors:** Regev Cohen, Talya Finn, Frida Babushkin, Yael Paran, Ronen Ben Ami, Alaa Atamna, Sharon Reisfeld, Gabriel Weber, Neta Petersiel, Hiba Zayyad, Eyal Leshem, Miriam Weinberger, Yasmin Maor, Nicola Makhoul, Lior Nesher, Galia Zaide, Dar Klein, Adi Beth-Din, Yafit Atiya-Nasagi

**Affiliations:** Laniado Hospital, Netanya, Israel (R. Cohen, T. Finn, F. Babushkin);; Technion–Israel Institute of Technology, Haifa, Israel (R. Cohen, T. Finn, S. Reisfeld, G. Weber);; Tel-Aviv Sourasky Medical Centre, Tel-Aviv, Israel (Y. Paran, R. Ben Ami);; Tel Aviv University, Tel Aviv (Y. Paran, R. Ben Ami, A. Atamna, E. Leshem, M. Weinberger, Y. Maor);; Rabin Medical Centre, Petah-Tikva, Israel (A. Atamna);; Hillel Yaffe Medical Centre, Hadera, Israel (S. Reisfeld);; Carmel Medical Centre, Haifa (G. Weber);; Rambam Medical Centre, Haifa (N. Petersiel);; Padeh Poriya Medical Centre, Tiberias, Israel (H. Zayyad);; Bar Ilan University, Safed, Israel (H. Zayyad, N. Makhoul); S; heba Medical Centre, Ramat Gan, Israel (E. Leshem);; Shamir (formerly Assaf Harofeh) Medical Center, Zerifin, Israel (M. Weinberger);; Edith Wolfson Medical Centre, Holon, Israel (Y. Maor);; Galilee Medical Centre, Nahariya, Israel (N. Makhoul); Soroka Medical Centre, Beer Sheba, Israel (L. Nesher);; Ben-Gurion University of the Negev, Beer Sheba (L. Nesher);; Israel Institute for Biological Research, Ness Ziona, Israel (G. Zaide, D. Klein, A. Beth-Din, Y. Atiya-Nasagi)

**Keywords:** R. conorii Israeli tick typhus strain, Rickettsia conorii israelensis, R. conorii Malish strain, Rickettsia conorii conorii, Rickettsia africae, spotted fever group rickettsiosis, epidemiology, Israeli spotted fever, Mediterranean spotted fever, Rhipicephalus sanguineus, ticks, vector-borne infections, zoonoses, Israel, bacteria

## Abstract

In a multicenter, nationwide, retrospective study of patients hospitalized with spotted fever group rickettsiosis in Israel during 2010–2019, we identified 42 cases, of which 36 were autochthonous. The most prevalent species was the *Rickettsia conorii* Israeli tick typhus strain (n = 33, 79%); infection with this species necessitated intensive care for 52% of patients and was associated with a 30% fatality rate. A history of tick bite was rare, found for only 5% of patients; eschar was found in 12%; and leukocytosis was more common than leukopenia. Most (72%) patients resided along the Mediterranean shoreline. For 3 patients, a new *Rickettsia* variant was identified and had been acquired in eastern, mountainous parts of Israel. One patient had prolonged fever before admission and clinical signs resembling tickborne lymphadenopathy. Our findings suggest that a broad range of *Rickettsia* species cause spotted fever group rickettsiosis in Israel.

Spotted fever group rickettsioses (SFGRs) are arthropodborne diseases caused by obligate intracellular, gram-negative bacteria of the genus *Rickettsia*. SFGRs are associated with ≈20 species of *Rickettsia*, of which 16 are considered human pathogens (*1*,*2*). Recent introduction of molecular methods provided more information about SFGR agents causing human disease and enabled their identification, for which the clinical significance of some remains lacking (*3*). *R. conorii* complex, the etiologic agent of Mediterranean spotted fever, includes 4 strains: *R. conorii* Malish (cause of Mediterranean spotted fever), *R. conorii* Astrakhan (cause of Astrakhan fever), *R. conorii* Indian tick typhu*s* (cause of Indian tick typhus), and *R. conorii* Israeli tick typhus strain (ITTS, cause of Israeli spotted fever [ISF]) (*4*–*7*).

ISF begins as fever followed by a maculopapular rash, usually involving the palms and soles and frequently accompanied by systemic symptoms. Most cases are self-limiting, but some may lead to organ failure and death. Clinical and epidemiologic presentations caused by other strains of rickettsiae may vary (*5*,*8*–*11*). Studies from Portugal indicate that compared with Mediterranean spotted fever, ISF is characterized by lower rates of eschar and tick-exposure history, higher frequency of gastrointestinal symptoms, and greater severity of illness with a high case-fatality rate (*10*,*12*,*13*). Case-fatality rates in Israel before 1998 were reportedly 0.7%, but incidence in some years (e.g., 1997) was higher (3.5%) (*14*). Since 1998, several case reports of fatal SFGR in Israel have been published (*9*,*15*–*17*), along with reports of 22 other patients with sepsis requiring hospitalization (*9*,*17*–*22*). Of these, isolates from only 3 patients were sequenced and identified as *R. conorii* ITTS; 2 of these patients exhibited purpura fulminans and all 3 died (*9*,*15*). Few studies of ISF have been conducted in Israel; most were conducted during the 1990s and relied on serologic diagnostic methods that cannot differentiate between the SFGRs (*23*). In Israel, SFGR is a notifiable disease, and in recent years, incidence has increased; 51 cases (including 7 deaths) were reported to the Israeli Ministry of Health in 2017, compared with the average yearly incidence of 26 cases during 2014–2016 (*24*).

The etiology of SFGR in Israel is thought to be *R. conorii* ITTS on the basis of limited molecular identification of this strain from clinical cases and from ticks (*9*,*15*,*25*). However, the yearly variations in disease severity and clinical manifestations are intriguing and may suggest involvement of >1 species of spotted fever group (SFG) *Rickettsia*. Using a large database from the national reference center, we studied the specific species of *Rickettsia* that cause SFGRs in Israel and characterized their unique epidemiology and clinical features. Institutional review board approval was granted at the principal investigator site (0002-19-LND) and for each participating institute.

## Methods

### Study Design

We conducted a multicenter, retrospective study of hospitalized patients with an SFGR during 2010–2019. The study included SFGR diagnosed by molecular methods at the Israel Institute for Biological Research (IIBR; Ness Ziona, Israel), which serves as the national reference center for *Rickettsia*. Blood and tissue samples from hospitalized patients with a suspected SFGR are occasionally submitted to the IIBR for molecular diagnosis, at the discretion of the treating physician. For cases of successful molecular identification, the referring medical center was requested to provide patient demographic and clinical data from the medical charts at each participating site. Deidentified data were integrated into a central database. 

### Serology and Molecular Diagnoses 

The IIBR tested serum samples for antibodies against *R. conorii* and *R. typhi* by an in-house immunofluorescence assay (cutoff for IgG of 1:100), as previously described (*26*). Skin biopsy samples, whole blood, cerebrospinal fluid, and other tissues were tested by PCR (*17*,*27*). Stored SFG-positive *Rickettsia* DNA samples and sequenced unique regions from 4 conserved *Rickettsia* genes were batch tested (primers listed in Table 1). *R. africae* was identified by real-time PCR targeting an internal transcribed spacer (*30*). These analyses enabled identification of ISF, *R. africae, R. conorii* Malish strain, and a new *Rickettsia* variant. Each set of reactions included a positive control and nontemplate as a negative control. The same primers were used for sequencing as for amplification. For species-level identification, we compared sequence results by using BLAST (https://www.ncbi.nlm.nih.gov/BLAST).

**Table 1:** Oligonucleotide primers used for PCR amplification and sequencing of *Rickettsia* species in study of spotted fever group rickettsioses in Israel, 2010–2019*

Primer	Target gene	Primer sequence, 5′ → 3′
213F	*OmpA*	AATCAATATTGGAGCCGGTAA
667R	*OmpA*	ATTTGCATCAATCGTATAAGTAGC
120F	*OmpA*	AAGGAGCTATAGCAAACGGCA
760R	*OmpA*	TATCAGGGTCTATATTCGCACCTA
760newF	*OmpA*	TAGGTGCGAATATAGACCCTGATA
1231R	*OmpA*	TGGCAATAGTTACATTTCCTGCAC
373F	*gltA*	TTGTAGCTCTTCTCATCCTATGGC
1138R	*gltA*	CATTTGCGACGGTATACCCATA
Rico173F	*gltA*	CGACCCGGGTTTTATGTCTA
1179R	*gltA*	TCCAGCCTACGATTCTTGCTA
gltA_EXT_R	*gltA*	TACTCTCTATGTACATAACCGGTG
gltA_NES_F	*gltA*	ATGATTGCTAAGATACCTACCATC
1497_R	*OmpB*	CCTATATCGCCGGTAATT
3462_F	*OmpB*	CCACAGGAACTACAACCATT
4346_R	*OmpB*	CGAAGAAGTAACGCTGACTT
607_F	*OmpB*	AATATCGGTGACGGTCAAGG
D1390R	*Sca4*	CTTGCTTTTCAGCAATATCAC
D767F	*Sca4*	CGATGGTAGCATTAAAAGCT

*All primers were designed at the Israel Institute for Biological Research (Ness Ziona, Israel), except Rico173F (*25*), rickettsial *OmpB* primers (*28*), and *sca4* primers (*29*).

### Mapping Locations of SFGR Acquisition 

We recorded the site of presumed rickettsiosis acquisition for autochthonous cases. When the site was unknown, we used the patient’s address. 

### Statistical Analyses

We used descriptive statistics to summarize patient characteristics. We calculated differences between categorical and continuous variables by using Pearson χ^2^, Student *t*, and Mann-Whitney tests, as appropriate. We used 1-way analysis of variance to analyze differences among groups. For statistical analyses, we used SPSS Statistics 25 (https://www.ibm.com). We considered 2-sided p<0.05 to be significant.

## Results

### SFGR Cases Diagnosed by IIBR in the Past Decade

**Figure 1:**
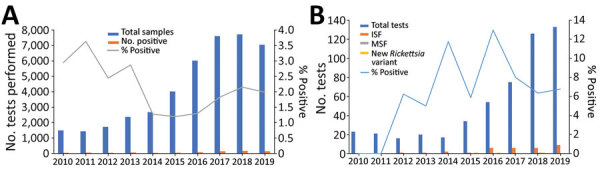
Spotted fever group rickettsioses, Israel, 2010–2019. A) Serologic tests performed in the Israeli central laboratory for rickettsiosis and number of positive cases per year. B) Autochthonous cases identified to the strain level. ISF, Israeli spotted fever; MSF, Mediterranean spotted fever.

In the 10-year study period, 1,985 cases of rickettsioses from the community and hospitals countrywide were diagnosed at IIBR by serologic testing; 811 were SFGR and 1,174 were murine typhus. Another 89 cases were positive by PCR, 66 for SFGR and 23 for murine typhus (Figure 1, panels A, B). Comparing 2010–2014 with 2015–2019 indicated an increased number of serologic tests performed yearly (from an average of ≈2,000 to ≈6,500). The percentage of positive serologic results decreased from 3% in 2010–2013 to 1.2% in 2014–2016 and then increased again to 2% in 2017–2019. Concomitantly, a 4-fold increase in total PCR tests performed was accompanied by a 7-fold increase in positive PCR results (average of 2.2–15.6 positive cases/year in the 2 periods). Of the 66 SFGR cases positive by PCR, 42 (64%) were identified to the strain level; for the other 24 cases, the classification failed, probably because of a low number of DNA copies in the original sample.

### *Rickettsia* Strains

The most prevalent *Rickettsia* strain in this study was *R. conorii* ITTS, found in 33/42 (79%) of cases identified to the strain level. The other strains accounted for 4 cases of *R. africae* infection, 2 of *R. conorii* Malish strain infection, and 3 of the newly identified *Rickettsia* variant (*27*). Most (36/42, 86%) cases were autochthonous: 32/36 (89%) were caused by *R. conorii* ITTS, 1 by *R. conorii* Malish strain, and the remaining 3 by the new *Rickettsia* variant (Figure 1, panel B). The 6 imported cases included 4 infections with *R. africae,* all acquired during a safari (in South Africa, Mozambique, Zimbabwe, or Botswana); 1 *R. conorii* ITTS imported from Cyprus; and 1 *R. conorii* Malish strain imported from New Delhi, India.

Molecular diagnosis was performed from skin biopsy samples from 27 patients (19 with ISF, 3 with new *Rickettsia* variant infection, 1 with *R. conorii* Malish strain infection, and 4 with African tick bite fever [ATBF]); from the blood of 19 patients (16 with ISF, 2 with *R. conorii* Malish strain, and 1 with new *Rickettsiae* variant); and from cerebrospinal fluid, lymph node, and liver biopsy postmortem samples from 1 patient with ISF. Seven patients were positive by PCR of samples from >1 source, mostly skin biopsy and whole blood. Serologic testing was performed for 36/42 (86%) patients; results were available for 26. Of these, results were negative for 16 (61%) and the median time between disease onset to last serology test was 6.2 days (range 0–11 days); for 10 (39%), the result was either positive (5 patients) or borderline (5 patients) and median time from disease onset to last serologic test 15.5 days (range 2–29 days).

### Geographic and Seasonal Distribution of Cases

**Figure 2:**
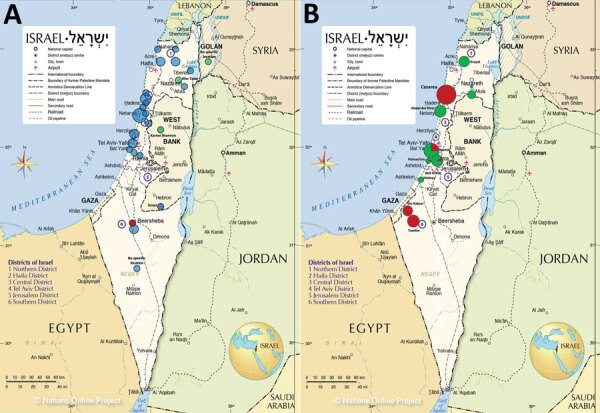
Geography of spotted fever group rickettsioses, Israel, 2010–2019. A) Presumed areas of autochthonous infection acquisition (n = 36 cases). B) Tick collection sites and tick species collected during 2014 by Rose et al. (*31*). ISF, Israeli spotted fever; MSF, Mediterranean spotted fever. Source: Nations Online Project (https://www.nationsonline.org).

The 36 autochthonous cases were reported from 12 hospitals representing most areas of Israel: 6 from central Israel, 4 from northern Israel, and 2 from southern Israel. *R. conorii* ITTS was reported from all but 1 hospital. All cases of *R. africae* infection were reported from 1 hospital. The most abundant concentration of cases (26/36, 72%, all *R. conorii* ITTS) was along the Mediterranean shoreline, with the highest aggregation of cases in the Sharon and Haifa districts (Figure 2, panel A). Ten cases were acquired inland, of which 6 were caused by *R. conorii* ITTS and 3 the new *Rickettsia* variant. Those 3 cases were presumably acquired in more mountainous areas of Israel. The *R. conorii* Malish strain was acquired in the desert region near Beer-Sheba.

Nearly half (27/61, 44%) of SFGR cases occurred during the 3 summer months, peaking in August. For the other seasons, 24% were recorded in the fall, 24% in spring, and 8% in winter.

### Patient Characteristics and Demographics

Median patient age was 50.5 years (interquartile range [IQR] 26–66 years), 25/42 (60%) were male, and 21/42 (50%) had >1 significant previous medical conditions (diabetes mellitus and dyslipidemia were the most prevalent, at 19% each) (Table 2). We found no significant differences between the 4 groups of rickettsial infections with regard to patient age, sex, and previous medical conditions. Although a history of tick exposure was rarely reported (2/42, 5% of patients, all in the ISF group), domestic animal exposure was quite common (reported by 25/42, 60% of patients), most commonly to dogs (20/42, 48%) and cats (5/42, 12%). Few patients reported exposure to cattle, sheep, or rats.

**Table 2:** Demographics and epidemiologic data for patients with spotted fever group rickettsiosis, according to rickettsial species, Israel, 2010–2019*

Patient data	*R. conorii* Israeli tick typhus strain	*R. conorii* Malish strain	*R. africae*	New *Rickettsia* variant	Total	p value
No. cases	33	2	4	3	42	
Age, y, median (IQR)	48 (18–64)	38	55.5 (52.5–64)	66	50.5 (26–66)	NS
Sex						
M	20 (61)	1 (50)	2 (50)	2 (67)	25 (60)	NS
F	13 (39)	1 (50)	2 (50)	1 (33)	17 (40)	NS
Any previous medical condition†	17 (51)	1 (50)	1 (25)	2 (66)	21 (50)	NS
Diabetes mellitus	6 (18)	1 (50)	0	1 (33)	8 (19)	NS
Dyslipidemia	7 (21)	0	0	1 (33)	8 (19)	NS
Hypertension	7 (21)	0	0	0	7 (17)	NS
Obesity	3 (9)	0	0	0	3 (7)	NS
COPD	1 (3)	0	1 (25)	1 (33)	3 (7)	NS
Chronic liver disease	1 (3)	1 (50)	0	0	2 (5)	NS
Alcohol abuse	3 (9)	0	0	0	3 (7)	NS
Psychiatric disorder/dementia	3 (9)	0	0	0	3 (7)	NS
Drug abuse	2 (6)	0	0	0	2 (5)	NS
Congestive heart failure	2 (6)	0	0	0	2 (5)	NS
Chronic renal failure	1 (3)	0	0	0	1 (2.5)	NS
Exposure history						
Tick	2 (6)	0	0	0	2 (5)	NS
Animals, species	20 (60), 17 dogs, 4 cats, 2 rats, 2 sheep, 1 cow)	0	2 (50),1 dog, 1 African safari	3 (100), 2 dogs, 1 cat	25 (59)	NS
Recent overseas travel	1 (3), to Cyprus	1 (50), to India	4 (100), to Africa	0	6 (14)	<0.0001

*Values are no. (%) unless otherwise indicated. COPD, chronic obstructive pulmonary disease; IQR, interquartile range; NS, not significant (p>0.05). †Includes all conditions described in the table except previous stroke, previous nonactive malignancy, atrial fibrillation, active malignancy, or immunosuppression.

### Clinical Features

All patients were hospitalized except for 1 with ATBF, who had mild disease (Table 3). The mean duration of stay was 11.9 days, and median (IQR) was 5 (3–10) days. Fever affected 41/42 (98%) patients. The mean number of days with fever until hospitalization was 5.2, and the median (IQR) was 5 (3–6) days; 98% of patients were hospitalized by day 9 of fever onset. For patients infected with the new *Rickettsia* variant, the mean interval was significantly longer (13.6 ± 13.3 days, range 5–29 days; p = 0.002 when compared with the other groups); 1 patient in this group had fever of unknown origin for 29 days before hospitalization.

**Table 3:** Clinical features of patients with spotted fever group rickettsiosis, according to rickettsial species, Israel, 2010–2019*

Patient data	*R. conorii* Israeli tick typhus strain	*R. conorii* Malish strain	*R. africae*	New *Rickettsia* variant	Total	p value
No. cases	33	2	4	3	42	
Fever	32 (97)	2 (100)	4 (100)	3 (100)	41 (98)	NS
Fever to admission interval, d, mean (± SD, range)	4.5 (± 2.1, 0–8)	6.5 (± 3.5, 4–9)	3.5 (± 2.4, 1–6)	13.6 (± 13.3, 5–29)	5.2 (± 4.3, 0–29)	0.002†
Systemic rash	33 (100)	1 (50)	0	3 (100)	37 (88)	<0.0001‡
Rash type						
Macular only	4 (12)	0	0	0	4 (10)	
Maculopapular	19 (58)	1 (100)	0	2 (67)	22 (53)	
Macular and petechial	5 (15)	0	0	1 (33)	6 (15)	
Petechial only	2 (6)	0	0	0	2 (5)	
Purpura fulminans	3 (9)	0	0	0	3 (7)	
Palm and sole involvement						
Yes	27 (82)	1 (100)	0	2 (67)	30 (73)	<0.0001‡
No	4 (12)	0	4 (100)	1 (33)	9 (22)	
Unknown	2 (6)	0	0	0	2 (5)	
Fever to rash interval, d, mean (± SD, range)§	3.2 (± 2.2, 0–8)	−1¶	NR	15 (± 16.9, 3−27)#	3.7 (± 4.6, 0–27)	
Eschar	4 (12)	1 (50)	4 (100)	1 (33)	10 (24)	0.011‡
>1 eschar	1 case (3 lesions)	None	None	None	None	
Location						
Lower limb	3	1	4	0	8	
Upper limb	1	0	0	0	1	
Neck	0	0	0	1	1	
Other signs/symptoms						
Lymphadenitis	2 (6)	1 (50)	1 (25)	1 (33)	5 (12)	NS
Lymphangitis	0	0	0	0	0	NS
Myalgia	13 (39)	2 (100)	1 (25)	2 (67)	18 (43)	NS
Arthralgia	5 (15)	0	0	1 (33)	6 (14)	NS
Cough	7 (21)	1 (50)	0	1 (33)	9 (21)	NS
Diarrhea	7 (21)	2 (100)	0	1 (33)	10 (24)	NS
Rigors	12 (36)	2 (100)	1 (25)	0	15 (36)	NS
Headache	14 (42)	2 (100)	1 (25)	1 (33)	18 (43)	NS
Photophobia	1 (3)	0	0	0	1 (2)	NS
Confusion	10 (30)	0	0	2 (67)	12 (28)	NS
Meningoencephalitis	8 (24)	0	0	0	8 (19)	NS
Fever of unknown origin	5 (15)	0	0	1 (33)	6 (14)	NS
ARDS	10 (30)	1 (50)	0	2 (67)	13 (31)	NS
DIC	10 (30)	1(50)	0	0	11 (26)	NS
Shock	13 (39)	2 (100)	0	1 (33)	16 (38)	NS
Hospitalization	33 (100)	2 (100)	3 (75)	3 (100)	41 (98)	NS
LOS, d, mean (± SD, range)	8.9 (± 10.4, 1–47)	12 (± 2.8, 10–14)	2.2 (± 2, 0–4)	57.6 (± 91, 5–163)	11.9 (± 25.6, 0–163)	0.01†
Intensive care admission	17 (52)	2 (100)	0	1 (33)	20 (48)	NS
Mechanical ventilation	13 (39)	2 (100)	0	1 (33)	16 (38)	NS
Death from rickettsiosis	10 (30)	0	0	0	10 (24)	NS**

*Values are no. (%) unless otherwise indicated. ARDS, acute respiratory distress syndrome; DIC, disseminated intravascular coagulation; LOS, length of stay; NS, not significant. †Between the new *Rickettsia* group and other spotted fever groups, using 1-way analysis of variance. ‡Between *R. africae* and the other 3 *Rickettsia* spotted fever groups, using Pearson χ^2^ tests. §Data available for 36 patients: 1 had no fever (Israeli spotted fever), 1 had no rash (*R. conorii Malish strain*), and 4 had unknown date of rash appearance (3 Israeli spotted fever strain and 1 the new species). ¶One *R. conorii* Malish patient had no rash and the other had rash before fever. #For 1 patient, the date of rash appearance was not documented. **p = 0.058 for *R. conorii* Israeli tick typhus strain compared with the other *Rickettsia* groups, using Pearson χ^2^ test.

Skin involvement in SFGR patients—either rash or eschar—was nearly universal (41/42, 98%). Systemic rash was documented for 37/42 (88%) of patients and absent for all 4 with ATBF, 1 with *R. conorii* Malish strain infection. The prevalent rash type was maculopapular (22/42, 53%), followed by macular and petechial (6/42, 15%) and macular (4/42, 10%). Involvement of palms and soles was common (30/38, 79%) for those with non-ATBF rickettsiosis. Purpura fulminans was seen in 3 patients, all within the ISF group (9% of patients in this group). Eschar was present on 12% patients with ISF, 100% with ATBF, 1 of the 2 with *R. conorii* Malish infection, and 1 of the 3 infected with the new *Rickettsia* variant. Except for 1 ISF patient who had 3 lesions, typically patients had only 1 eschar, usually on the lower limbs, including those in the ATBF group.

The time interval between fever onset and appearance of rash differed among the groups: for ISF patients, the mean (range) was 3.2 (0–8) days, and for patients infected with the new *Rickettsia* variant, the mean and median were 15 days. Of the 2 patients with *R. conorii* Malish strain infection, 1 had rash that reportedly appeared 1 day before fever onset.

Systemic symptoms (e.g., myalgia and headache) were common for all patients with non-ATBF *Rickettsia* infection, and meningoencephalitis was evident in 8/33 (24%) of patients with ISF. For ATBF patients, rates of systemic symptoms were lower. Similarly, about one third of patients with non-ATBF rickettsiosis but none with ATBF experienced severe disease with shock, multiorgan failure, need for mechanical ventilation, disseminated intravascular coagulation, acute respiratory distress syndrome, and need for intensive care. 

### Mortality Rate and Related Risk Factors

Of the 42 patients, 10 (23.8%) died during their hospital stay, all within 20 days of admission (mean ± SD [range] 6.3 ± 6.3 [1–20] days, median [IQR] was 3.5 [2.5–11] days). Mean age among those who died was 55 (14–95) years and among those who survived, 42.5 (1–83) years (p = 0.8). Mortality rate was remarkably high for the ISF group, reaching 30%, although none in the other 3 groups died. The only patient-related risk factor significantly associated with death was alcohol abuse (hazard ratio [HR] 5.8, 95% CI 1.14–30.4). Disease-related risk factors associated with death were hemodynamic shock at admission (HR 10.7, 95% CI 1.33–87.3), disseminated intravascular coagulation (HR 4.7, 95% CI 1.19–18.6), and jaundice (HR 6.7, 95% CI 1.4–32.2). All patients in our study received doxycycline during hospitalization; the mean ± SD (range) interval from fever onset to doxycycline receipt was 6 ± 4.3 (1–29) days. This interval did not differ between groups of patients who survived (5.7 ± 4.8 days) or died (6.9 ± 1.7 days) (p = 0.4).

### Laboratory Data`

Except for ATBF, laboratory findings did not differ between the groups (Table 4). During the first 3 days of hospitalization, acute kidney injury was common (>50%); other common findings included hepatic injury accompanied with mild to moderate jaundice, mild rhabdomyolysis, mild international normalized ratio prolongation, thrombocytopenia, and lymphocytopenia. Leukocytosis was more common than leukopenia, and C-reactive protein levels were >100 mg/L (reference <5 mg/L). ATBF cases were distinctly different and patients showed much milder systemic reactions: no hepatocellular injury, jaundice, rhabdomyolysis, or thrombocytopenia; and significantly lower C-reactive protein levels (mean 35 mg/L; p = 0.034) (Table 4).

**Table 4:** Laboratory features of 42 patients with spotted fever group rickettsiosis according to rickettsial species, during hospitalization days 1–3, Israel, 2010–2019*

Feature†	*R. conorii *Israeli tick typhus strain	*R. conorii *Malish strain	*R. africae*	New *Rickettsia* variant	Total	p value
No. cases	33	2	4	3	42	
Acute kidney injury (creatinine >1.3 mg/dL), no. (%)	20/32 (62.5)	0/2	1/4 (25)	1/3 (33)	22/41 (54)	NS‡
Creatinine, mg/dL, mean (range)	1.8 (0.13–6.25)	0.83 (0.7–0.9)	0.96 (0.6–1.3)	1.6 (1.17–2.4)	1.6 (0.1–6.25)	NS§
Hepatocellular injury pattern						
AST or ALT >2 ULN, no. (%)	24/33 (73)	2/2 (100)	0/4 (100)	2/3 (67)	28/42 (67)	0.02‡
AST, IU/L, mean (range)	855 (42–8,895)	249 (270–228)	27 (23–31)	83 (60–107)	728 (23–8,895)	NS§
ALT, IU/L, mean (range)	334 (12–2,881)	94 (109–80)	26 (25–29)	88 (40–164)	22/41 (54)	NS§
Cholestatic injury pattern						
Alkaline phosphatase or GGT >2 ULN, no. (%)	16/32 (48)	2/2 (100)	1/4 (25)	2/3 (67)	21/41 (51)	NS‡
Alkaline phosphatase, IU/L, mean (range)	196 (32–1,056)	109 (67–152)	64 (63–65)	265 (60–416)	190 (32–1,056)	NS§
GGT, IU/L, mean (range)	156 (15–1,026)	150 (129–171)	73 (21–125)	507	154 (15–1,026)	NS§
Jaundice, bilirubin >1.3 mg/dL, no. (%)	14/33 (42)	1/2 (50)	0/3	0/3	15/41 (36)	NS‡
Bilirubin, mg/dL, mean (range)	1.77 (0.29–10)	2.6 (1.3–4)	0.4 (0.3–0.5)	0.93 (0.6–1.3)	1.46 (0.29–10)	NS§
C-reactive protein >5 mg/L, no. (%)	30/30 (100)	1/1 (100)	4/4 (100)	3/3 (100)	38/38 (100)	NS‡
C-reactive protein, mg/L, (range)	207 (17–460)	102	35 (17–61)	223 (131–273)	187 (17–410)	0.034§
Rhabdomyolysis, creatine kinase >ULN, no. (%)	17/30 (52)	1/2 (50)	0/2	0/3	18/37 (49)	NS‡
Creatine kinase, IU/L (range)	1,345 (81–8,900)	271 (128–414)	92 (71–113)	79 (57–102)	1,119 (57–8,900)	NS§
Complete blood count						
Leukocytosis, >10,000 leukocytes/μL, no. (%)	15/33 (45)	0/2	0/4	1/3 (33)	16/42 (38)	NS‡
Leukopenia, <4,000 leukocytes/μL, no. (%)	6/33 (18)	1/2 (50)	0/4	1/3 (33)	8/42 (19)	NS‡
Leukocytes, × 10^3^/μL, mean (range)	14.2 (2.5–43.3)	4.5 (2.6, 6.4)	4.5 (4–5.2)	10.2 (3.9–17.1)	13.1 (2.5–43.3)	NS§
Lymphocytopenia, ALC <1,500/μL, no. (%)	30/33 (91)	2/2 (100)	4/4 (100)	3/3 (100)	36/42 (93)	NS‡
ALC, × 10^3^/μL, mean (range)	0.9 (0.2–6.9)	0.35 (0.3–0.4)	1.26 (1.2–1.3)	1.1 (0.6–2.3)	0.9 (0.2–6.9)	NS§
Thrombocytopenia, platelets <150K cells/μL, no. (%)	29/32 (88)	2/2 (100)	0/4	3/3 (100)	34/41 (83)	0.001‡
Platelets, × 10^3^/μL, mean (range)	84 (15–271)	36 (26–46)	238 (164–316)	64 (37–101)	82 (15–271)	0.001§
Coagulopathy, INR >1.2, no. (%)	16/33 (48)	1/2 (50)	0/2	0/3	17/40 (42)	NS‡
INR, mean (range)	1.38 (0.9–3)	1.5 (1.1–1.9)	0.96 (0.93–1)	1.09 (1.04–1.16)	1.36 (0.9–3)	NS§
Molecular diagnosis source, no. (%)						
Skin biopsy sample/eschar	19 (58)	1 (50)	4 (100)	3 (100)	27 (64)	
Blood	16 (48)	2 (100)	0	1 (33)	19 (45)	
CSF	1 (3)	0	0	0	1 (2)	
Other organs	2 (6)¶	0	0	0	2 (5)	
Serologic diagnosis, no. (%) samples	32 (97)	2 (100)	1 (25)	1 (33)	36 (86)	
Positive	5 (16)	0	0	0	5 (14)	
Borderline	2 (6)	0	0	1 (33)	3 (8)	
Negative	25 (78)	2 (100)	1 (25)	0	28 (78)	

*ALC, absolute lymphocyte count; ALT, alanine aminotransferase; AST, aspartate aminotransferase; CSF, cerebrospinal fluid; GGT, gamma glutamyl transpeptidase; INR, international normalized ratio; NS, not significant; ULN, within upper limit of normal range. †Highest or lowest levels reached within the time frame are reported. ‡By Pearson χ^2^ test. §By 1-way analysis of variance, conducted on day-of-admission data. ¶One from liver biopsy sample and 1 from enlarged lymph node.

### New *Rickettsia* Variant

In 3 epidemiologically unrelated patients, we identified a new *Rickettsia* variant of the ISF clade. Partial sequencing of the following conserved *Rickettsia* genes indicated that the isolates were 100% identical to each other: *glt*A (GenBank accession no. MW366541), *rOmpA* (MW366542), and *sca4* (MW366543). Highest similarity (92.2%–96.9% homology) was seen with *R. conorii* Astrakhan*, R. slovaca, R. sibirica*, and *R. conorii* ITTS. Phylogenetic analysis could not assign the new variant to any existing strain, as previously described (*27*). The 3 cases were unrelated spatially or temporally (Appendix).

## Discussion

With this nationwide clinical and molecular study, we provide molecular evidence that *R. conorii* ITTS is the most commonly identified strain of SFG *Rickettsia* among hospitalized patients in Israel. Although SFGR is a reportable disease in Israel, it is underreported; of the average of 81 cases diagnosed by serologic testing each year according to the IIBR laboratory data, an average of 27 cases are reported to the Ministry of Health each year (*24*) (Figure 1, panel A). Because we lack clinical information for many of these cases, such as severity of illness and hospitalization, we can only partially infer the role of this strain in causing SFGR among hospitalized and ambulatory patients. Although *R. conorii* ITTS was suspected as the causative agent of SFGR in Israel, this suspicion has been supported only by very limited data: rare case reports of fatal human cases (*9*,*15*) and a few studies of ticks (*25*,*32*). 

Testing for spotted fever by serology and recently by PCR increased during 2017–2019 compared with the preceding 3 years. This rise probably represents increased clinical suspicion of SFGR and a true increase in disease activity. Despite missing molecular data for ambulatory patients with mild cases of SFGR, we believe that during the past 3 years, *R. conorii* ITTS has led a silent outbreak of SFGR in Israel. Similarly, an unprecedented 46% increase in SFGR was reported during 2016–2017 by the US Centers for Disease Control and Prevention from states where SFGR is known to be endemic (*33*). US authorities were faced with the dilemma of whether this increased incidence should be ascribed to a true increase, increased testing, or inappropriate use of a single serologic test instead of paired tests, reflecting past infection in a disease-endemic area.

We report the epidemiologic features of SFGR patients, although comparison between *R. conorii* ITTS with the other groups was limited because of small numbers. In Israel, SFGR affects mainly young adults and has a mild predilection for men. SFGR has been endemic to the Sharon and Haifa Districts since the 1990s (*34*); in our study, these 2 districts accounted for 44% (16/36) of all autochthonous cases. These results are similar to those reported in 2014 by Rose et al., who also investigated the association between geographic data and SFGR-positive ticks. Areas with SFG *Rickettsia*–infected ticks were associated with brown-type soil, higher land surface temperatures, and higher precipitation (*31*). We observed a wide geographic distribution of human ISF cases with aggregation in northern Israel.

Although *R. conorii* ITTS seems to be the main *Rickettsia* causing clinical disease in Israel, why this strain is rarely found in ticks collected from Israel (*25*,*35*) and other countries (*7*,*36*,*37*) remains unclear. Studies from Israel have found *Rickettsia massiliae* to be more prevalent (≈10 fold) than *R. conorii* ITTS among questing ticks and ticks feeding on animals (*31*,*35*,*38*). This discrepancy could be explained by underreporting of *R. massiliae* infection in humans with mild or subclinical disease. Most patients in our study were hospitalized with severe disease and may represent a reporting bias of *R. conorii* ITTS, which causes more severe disease. Patients with milder illness, potentially caused by other rickettsiae, may not be hospitalized, and illness may resolve undiagnosed, without the need for molecular studies.

Rose et al. (*31*) collected *Rhipicephalus sanguineus* and *Rh. turanicus* ticks from geographic locations similar to the presumed areas of the clinical autochthonous cases in our study (Figure 2, panel B). However, *R. conorii* ITTS was found only rarely (1.8%) and strictly in *Rh. sanguineus* ticks. Hence, the role of *Rh. turanicus* ticks as possible vectors of *R. conorii* ITTS and *R. massiliae* as a cause of rickettsiosis in humans should be further explored. Although clearly reported as a cause of SFGR, *R. massiliae* is still rarely isolated from human patients (*39*). Deleterious effects of *R. conorii* on *Rh. sanguineus* tick fitness, resulting in infected ticks not surviving the winter, may explain its low prevalence among ticks in nature (*40*,*41*).

The new *Rickettsia* variant was found in the eastern and more mountainous parts of the country: the Golan Heights, the Galilee region, and the West Bank of the Palestinian Authority. This distribution may suggest a geographic niche for either this new *Rickettsia* or its vector and should be further explored in studies of tick collections from these mountainous areas. The single case of *R. conorii* Malish strain infection acquired locally in this study was in a 50-year-old man from the desert area, who had a necrotic eschar in the thigh and severe systemic disease.

Most cases were reported during the summer and peaked in August. This finding is consistent with previous reports (*12*) and may be attributed to increased activity of the vectors and to the aggressiveness and host indiscrimination of *Rh. sanguineus* ticks when exposed to higher temperatures (*42*).

Patients rarely remembered seeing or being bitten by ticks (only 2 remembered); however, exposure to animals was common (25/42, 59% of cases), mainly to dogs (20/25, 80%), the principal hosts of *Rh. sanguineus* ticks (the main reservoir of *R. conorii*). This finding implies that exposure to domestic pets is more relevant than exposure to ticks.

The clinical and laboratory features for patients in our case series were typical of SFGR, although eschar, which is considered rare in patients with ISF, was seen in 12% of patients, 1 of whom had 3 lesions. ISF caused purpura fulminans in 9% and meningoencephalitis in 24%. About half of ISF patients experienced multiorgan involvement that included kidney and liver injury, jaundice, rhabdomyolysis/myositis, and coagulopathy. Severe disease requiring intensive care was strikingly common (52%), and 30% of ISF patients died in hospital. The high mortality rate, previously reported for ISF infection (*10*), contrasted with lower rates from historical reports from Israel during the 1990s. This discrepancy may result from reporting bias with increased awareness in recent years, as well as improved laboratory capabilities. An additional possibility is an outbreak of a more virulent strain, such as *R. conorii* ITTS. Risk factors for death included only alcohol abuse, as previously described (*12*). Admission-to-treatment (doxycycline) interval was not significant.

The small number of cases in this investigation makes drawing conclusions or comparisons difficult; however, the new *Rickettsia* variant may lead to prolonged fever before care seeking and may resemble tickborne lymphadenopathy usually related to *Rickettsia slovaca* or *Rickettsia raoultii* (*11*). Patients with ATBF had a distinct clinical syndrome of a milder clinical disease; for 25%, systemic symptoms were limited to fever, myalgia, and headache with no systemic rash.

In conclusion, we report a nationwide case series of hospitalized patients with molecularly diagnosed SFGR over a decade in Israel, of which *R. conorii* ITTS was the principal cause of severe disease, multiorgan failure, and high mortality rates. We also describe a new *Rickettsia* variant, which may be associated with unique epidemiologic and clinical features. This study suggests that a broader range of species causes SFGR in Israel and that this possibility should be explored in larger, prospective studies, especially in light of the potential candidates found in ticks.

Appendix Supplemental information for 3 patients infected with new *Rickettsia* variant of the Israeli spotted fever clade.
