# Supplementary material for: Spotted Fever Group Rickettsioses in Israel, 2010–2019
Source: Emerg Infect Dis. 2021 Aug;27(8):2117–26. doi: 10.3201/eid2708.203661 (PMC8314820; doi:10.3201/eid2708.203661)
Supplement: Appendix — Supplemental information for 3 patients infected with new Rickettsia variant of the Israeli spotted fever clade. [file 20-3661-Techapp-s1.pdf]

Article DOI: https://doi.org/10.3201/eid2708.203661

Spotted Fever Group Rickettsioses in Israel, 2010–2019

Appendix

Patient 1 was a healthy 54-year-old man who had a prolonged fever of 27 days, after which a maculopapular rash not involving palms and soles appeared. On admission he had an eschar on his neck with local lymphadenopathy but no lymphangitis. He had systemic symptoms that included myalgia, arthralgia, cough, headache, and bilateral interstitial infiltrates on chest X ray. He had elevated CRP levels (~130mg/L), leukopenia, lymphopenia and thrombocytopenia but no kidney or liver injury. He received doxycycline on admission and had an uneventful hospitalization of 5 days. He lived in Kfar Tavor, located in the lower Galilee region, and owned a dog.

Patient 2 was a 66-year-old female nurse with diabetes mellitus and hyperlipidemia, who presented with 5 days of fever, sore throat, myalgia, vomiting and anorexia. A maculopapular rash appeared on day 3 of the fever. Doxycycline was administered intravenously upon admission, but she developed AKI, severe thrombocytopenia, liver injury, shock, ARDS and was admitted to the ICU. She developed encephalopathy and extremity necrosis that led to below-knee amputation of one leg. She was discharged after 163 days in hospital. She lived in the center of Israel but travelled to the Golan heights in the 2 weeks prior to her illness. She also owned a dog.

Patient 3 was a 75-year-old man with chronic obstructive lung disease, who presented with 7 days of fever, undated macular and petechial rash involving the palms and soles, and confusion. He did not have any other systemic symptoms. He had mild hepatocellular and cholestatic liver enzymes disturbance, severe thrombocytopenia (45,000 cells/mcl) and elevated CRP (273mg/L). He received doxycycline on admission and was discharged after 5 days. He lived in the Shomron area and owned a cat.
